# Impact of different pack sizes of paracetamol in the United Kingdom and Ireland on intentional overdoses: a comparative study

**DOI:** 10.1186/1471-2458-11-460

**Published:** 2011-06-10

**Authors:** Keith Hawton, Helen Bergen, Sue Simkin, Ella Arensman, Paul Corcoran, Jayne Cooper, Keith Waters, David Gunnell, Navneet Kapur

**Affiliations:** 1 Centre for Suicide Research, Department of Psychiatry, University of Oxford, Warneford Hospital, Oxford OX3 7JX, UK; 2 National Suicide Research Foundation, 1 Perrott Avenue, Cork, Ireland; 3 Centre for Suicide Prevention, University of Manchester, 2nd Floor, University Place, Oxford Road, Manchester M13 9PL, UK; 4 Derbyshire Healthcare NHS Foundation Trust, Level 2 Rehabilitation Centre, Royal Derby Hospital, Uttoxeter Road, Derby DE22 3NE, UK; 5 Department of Social Medicine, University of Bristol, Canynge Hall, 39 Whatley Road, Bristol BS8 2PS, UK

## Abstract

**Background:**

In order to reduce fatal self-poisoning legislation was introduced in the UK in 1998 to restrict pack sizes of paracetamol sold in pharmacies (maximum 32 tablets) and non-pharmacy outlets (maximum 16 tablets), and in Ireland in 2001, but with smaller maximum pack sizes (24 and 12 tablets). Our aim was to determine whether this resulted in smaller overdoses of paracetamol in Ireland compared with the UK.

**Methods:**

We used data on general hospital presentations for non-fatal self-harm for 2002 - 2007 from the Multicentre Study of Self-harm in England (six hospitals), and from the National Registry of Deliberate Self-harm in Ireland. We compared sizes of overdoses of paracetamol in the two settings.

**Results:**

There were clear peaks in numbers of non-fatal overdoses, associated with maximum pack sizes of paracetamol in pharmacy and non-pharmacy outlets in both England and Ireland. Significantly more pack equivalents (based on maximum non-pharmacy pack sizes) were used in overdoses in Ireland (mean 2.63, 95% CI 2.57-2.69) compared with England (2.07, 95% CI 2.03-2.10). The overall size of overdoses did not differ significantly between England (median 22, interquartile range (IQR) 15-32) and Ireland (median 24, IQR 12-36).

**Conclusions:**

The difference in paracetamol pack size legislation between England and Ireland does not appear to have resulted in a major difference in sizes of overdoses. This is because more pack equivalents are taken in overdoses in Ireland, possibly reflecting differing enforcement of sales advice. Differences in access to clinical services may also be relevant.

## Background

Paracetamol is commonly used in intentional self-poisoning [1]. Overdoses of paracetamol may result in liver damage, which can be fatal. As a result of a large number of deaths and liver transplants each year due to hepatotoxicity resulting from paracetamol overdose in the UK [2-4], legislation came into force in September 1998 to restrict pack sizes of paracetamol (and aspirin and their compounds) sold over the counter to a maximum of 32 tablets in pharmacies and 16 in non-pharmacy outlets [5]. Labelling and other packaging changes occurred around the same time.

In Ireland, similar legislation was introduced in October 2001 [6] but pack sizes were restricted to considerably lower maximum amounts than in the UK, namely a maximum pack size of 24 tablets in pharmacies and 12 tablets in non-pharmacy outlets, with just a single pack to be supplied in any one transaction.

The UK legislation appears to have had beneficial effects, at least in England and Wales, in terms of reduced sizes of overdoses and numbers of deaths and liver transplantations [1,7], although some commentators have disputed this [8-10]. These effects were not seen in Scotland [11]. In Ireland, on the basis of calls to the National Poisons Centre, the change in pack sizes of paracetamol appeared to have resulted in smaller overdoses (i.e. reduced number of tablets) in the first two years after the legislation [12]. Data on deaths involving paracetamol in Ireland have not been published.

In the UK, despite the significant reduction in number of deaths due to paracetamol-induced hepatoxicity [1], the death toll remains high. The annual number of poisoning deaths involving paracetamol alone which received a verdict of suicide, undetermined or accidental death registered during 2000 - 2008 varied between 90 and 155. In addition there are considerable numbers of deaths involving paracetamol compounds and paracetamol taken with other drugs [13-15].

Some authors have suggested that the maximum pack size for pharmacy sales in the UK should have been set at a lower level, more akin to the maximum pack size in Ireland [1,16]. Support for this comes from toxicological studies in which ingestion of 15 grams of paracetamol (i.e. 30 tablets) appeared to be associated with considerably elevated risk of hepatotoxicity [17]. A smaller maximum pack size might therefore have introduced a safety margin.

In order to provide information relevant to the question of whether it might be beneficial to further reduce pack sizes of paracetamol in the UK, we have conducted an investigation in which we have compared sizes of overdoses of paracetamol taken in the UK and in Ireland. We have used data from the Multicentre Monitoring of Self-harm project in England [18,19] and the National Deliberate Self-harm Registry in Ireland [20].

## Methods

We investigated the number of tablets of paracetamol consumed in overdoses which resulted in presentation between 2002 and 2007 to six general hospitals in three centres in England, and all general hospitals in Ireland, for persons aged 10 years or more. Data were restricted to non-fatal intentional self-poisoning episodes in which paracetamol was the sole medicinal agent consumed (with or without co-ingestion of alcohol), and where at least four tablets were taken at one time (i.e. double the maximum recommended single therapeutic dose of two tablets).

### Data sources

#### Multicentre Monitoring of Self-harm in England Project

This project is based on monitoring of general hospital presentations for intentional self-poisoning and self-injury in six major general hospitals in England [18,19], one in Oxford, three in Manchester and two in Derby. The study is coordinated by the Centre for Suicide Research at Oxford University. Information is collected on all presentations for intentional self-poisoning to the general hospitals, and includes (among other factors) gender, age, drugs used for self-poisoning, numbers of tablets, and consumption of alcohol during the six hours prior to self-poisoning and as part of the overdose. The information about numbers of tablets taken in overdose is usually based on patient self-report, but often corroborated by accounts of relatives and other informants and inspection of medicine containers.

#### National Registry of Deliberate Self Harm in Ireland

Information is collected on all presentations for intentional self-poisoning and self-injury to general hospitals in Ireland [20]. Information used in this study is collected on the same items as in the Multicentre Study of Self-harm in England, except that alcohol consumption related to self-poisoning is just recorded as a single variable (alcohol involved at the time of overdose).

### Ethical Approval

The monitoring systems in Oxford and Derby have approval from local Health/Psychiatric Research Ethics Committees to collect data on self-harm for local and multicentre projects. Self-harm monitoring in Manchester is part of a clinical audit system, and has been ratified by the local Research Ethics Committee. All three monitoring systems are fully compliant with the Data Protection Act of 1998. All centres have approval under Section 251 of the NHS Act 2006 (formerly Section 60, Health and Social Care Act 2001) to collect patient identifiable information without patient consent. The National Registry of Deliberate Self Harm in Ireland has ethical approval from the National Research Ethics Committee of the Faculty of Public Health Medicine, and from the relevant hospitals and Health Service Executive ethics committees.

### Data Analysis

Analyses comparing the number of tablets consumed, and number of packs used in overdoses of paracetamol in England and Ireland were conducted using the Chi-square, Mann-Whitney U and Kruskal Wallis tests. Analyses were done on combined data, and separately by gender, age group and alcohol involvement with the overdose (data from the English study on alcohol consumed during the six hours before self-poisoning and/or as part of the overdoses were combined for this analysis).

The number of packs used was calculated as a multiple of the non-pharmacy maximum pack size in each country, from 1 pack to 9 or more packs (e.g. in England an overdose involving up to 1 pack was 4-16 tablets; 2 packs was 17-32 tablets; 3 packs was 33-48 tablets, and so on. In Ireland, up to 1 pack was 4-12 tablets; 2 packs 13-24 tablets, 3 packs 25-36 tablets, and so on).

## Results

### Study samples

During the six-year study period 2002 to 2007 there were 31,107 hospital presentations for self-poisoning (alone) in the three English centres (six hospitals) and 42,877 (40 hospitals - 34 in 2002, 37 in 2003, 38 in 2004-2005, and 40 in 2006-2007) in Ireland. Of these, paracetamol was involved in 10,208 (32.8%) episodes in the English centres and 9057 (21.1%) in Ireland. Paracetamol alone (with or without alcohol) was involved in 5444 episodes in the English centres and in 3886 in Ireland.

Data on number of tablets taken in overdose was missing for 559 (10.3%) episodes in the English centres and 358 (9.2%) in Ireland. We also excluded episodes where fewer than 4 tablets were taken, where gender was missing, and where age was missing, or patients were under 10 years (totalling 27 (0.5%) in the English centres and l9 (0.5%) in Ireland). Thus the samples for inclusion in the study consisted of 4858 episodes in the English centres and 3509 in Ireland.

The female: male gender ratio for episodes was somewhat larger in the Irish patients (2.1:1) than in England (1.8:1) (Table 1). The age distributions of the two samples showed greater proportions of younger individuals in the Irish patients and older people in the English patients. Alcohol use at the time of overdose occurred with similar frequency in the English and Irish patients.

**Table 1:** Episodes of self-poisoning with known number of paracetamol tablets in England and Ireland 2002-2007

	*England N = 4858*	*Ireland N = 3509*	*Chi sq*	*P*
Gender				
Male	1736 (35.7%)	1146 (32.7%)		
Female	3122 (64.3%)	2363 (67.3%)	8.537	0.003
Age group				
10-24 y	2186 (45.0%)	1682 (47.9%)		
25-34 y	962 (19.8%)	753 (21.5%)		
35+ y	1710 (35.2%)	1074 (30.6%)	19.442	<0.001
Alcohol use				
Yes	1851 (38.1%)	1259 (35.9%)		
No or not known	3007 (61.9%)	2250 (64.1%)	4.311	0.038

Episodes of self-poisoning with known number of paracetamol tablets for persons aged 10 years or more in three centres (six general hospitals) in England, and in Ireland, 2002 to 2007

### Number of tablets taken in overdoses

**Figure 1:**
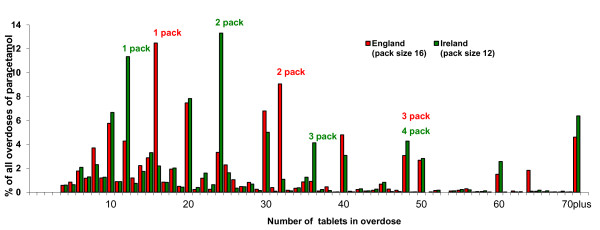
**Distribution of tablets taken in paracetamol overdose in England and Ireland**. Distribution of tablets taken in paracetamol overdose by persons aged 10 years or more, in England (three centres; six general hospitals) (N = 4858), and in Ireland (N = 3509), 2002 to 2007, shown as percentage of total number of overdoses of paracetamol in each country.

The pattern of the numbers of tablets taken in overdoses in the English centres and in Ireland is shown in Figure 1. There were clear peaks in each of the countries corresponding to the pack size limits for non-pharmacy and pharmacy sales. Thus in the English sample there were peaks at 16 and 32 tablets and in the Irish sample at 12 and 24 tablets. There were also peaks at multiples of these pack sizes. There were, in addition, peaks in both samples at 10 and multiples of 10 tablets.

The median number of tablets consumed in paracetamol overdoses did not differ significantly between the English (median 22 tablets) and Irish (median 24 tablets) patients (Table 2).

**Table 2:** Paracetamol tablets taken in overdose in England and Ireland 2002-2007 by gender and age group

	*England N = 4858*		*Ireland N = 3509*		*Mann Whitney*	
	*median*	*(IQR)*^*1*^	*median*	*(IQR)*^*1*^	*Z*	*P*
Both genders	22	(15-32)	24	(12-36)	-0.758	0.449
						
Males	28	(16-40)	27	(18-48)	-2.015	0.044
Females	20	(13-32)	20	(12-30)	-1.651	0.099
						
10-24 y	19	(12-32)	20	(12-30)	-1.179	0.239
25-34 y	24	(16-40)	24	(15-45)	-1.509	0.131
35+ y	26	(16-40)	24	(12-40)	-2.862	0.004
						
Males						
10-24 y	22	(15-32)	24	(14-36)	-1.364	0.173
25-34 y	30	(16-45)	30	(20-50)	-2.128	0.033
35+ y	30	(16-48)	30	(20-49)	-1.083	0.279
Females						
10-24 y	16	(12-30)	20	(12-28)	-0.655	0.513
25-34 y	20	(14-32)	24	(12-36)	-0.543	0.587
35+ y	24	(16-32)	23	(12-32)	-3.843	<0.001

^1 ^IQR = interquartile range

Number of tablets of pure paracetamol taken alone in overdose in England (three centres, six general hospitals), and in Ireland, 2002 to 2007, by gender and age group

In both countries the number of tablets taken differed significantly by gender, with larger overdoses in males than females (England, Kruskall Wallis Z = 150.882, P < 0.001; Ireland, Z = 71.052, P < 0.001). There was also a difference by age group, with larger overdoses being taken by older people in both males (England, Z = 48.948, P < 0.001; Ireland, Z = 36.555, P < 0.001) and females (England, Z = 68.973, P < 0.001; Ireland, Z = 22.165, P < 0.001).

When the samples were divided into three age groups and the two countries compared a small but nevertheless statistically significant difference in the median number of paracetamol tablets consumed between the English (median 24) and Irish (median 23) samples was found in age group 35 years and over (Table 2). This difference was confined to females.

### Involvement of alcohol in overdoses

In both England and Ireland, when alcohol was known to be involved in overdoses more paracetamol tablets tended to be taken (England, Z = -3.141, P = 0.002; and Ireland, Z = -2.105, P = 0.035). This pattern was found in males, but not females, in both countries (Table 3). There was a non-significant trend for smaller overdoses in males in Ireland who had not consumed alcohol compared with those in England.

**Table 3:** Number of paracetamol tablets taken in overdose in England and Ireland 2002-2007 by alcohol use

	*England N = 4858*		*Ireland N = 3509*		*Mann Whitney*	
	*median*	*(IQR)*^*1*^	*median*	*(IQR)*^*1*^	*Z*	*P*
Both genders						
Alcohol	24	(16-35)	24	(13-36)	-0.619	0.536
No/Not known	20	(14-32)	24	(12-36)	-0.328	0.743
						
Males						
Alcohol	30	(16-40)	30	(18-48)	-1.086	0.277
No/Not known	28	(16-40)	25.5	(16-48)	-1.739	0.082
						
Females						
Alcohol	20	(14-32)	20	(12-30)	-1.496	0.135
No/Not known	20	(13-32)	20	(12-30)	-0.954	0.340

^1 ^IQR = interquartile range

Number of tablets of pure paracetamol taken alone in overdose in England (three centres; six general hospitals), and in Ireland, 2002 to 2007, by alcohol use at the time or prior to the overdose

### Number of packs used in overdoses

When the numbers of tablets consumed in overdoses by individuals in each country were categorised into number of pack equivalents used (based on the non-pharmacy maximum pack size in each country, i.e. 16 tablets in England and 12 in Ireland), the mean number of packs used was greater in Ireland (2.63, 95% CI = 2.57-2.69) than England (2.07, 95% CI 2.03-2.10). The largest proportion of overdoses involved one pack only in England (39.0%), and three or more packs in Ireland (37.9%) (Table 4). Females and the younger age group (15-24 years) were more likely to use one pack in England and two packs in Ireland. Males and individuals in older age groups (25-34, 35+ years) were far more likely to use three or more packs in Ireland than in England.

**Table 4:** Number of paracetamol pack equivalents used in overdose in England and Ireland 2002-2007

	*England*	*Ireland*	*Chi square*	*P*
	*N (%)*	*N (%)*		
Both genders				
1 pack	1893 (39.0)	949 (27.0)		
2 packs	1790 (36.8)	1229 (35.0)		
3+ packs	1175 (24.2)	1331 (37.9)	215.6	<0.001
Males				
1 pack	521 (30.0)	217 (18.9)		
2 packs	638 (36.8)	327 (28.5)		
3+ packs	577 (33.2)	602 (52.5)	109.8	<0.001
Females				
1 pack	1372 (43.9)	732 (31.0)		
2 packs	1152 (36.9)	902 (38.2)		
3+ packs	598 (19.2)	729 (30.9)	135.6	<0.001
Age 15-24				
1 pack	1019 (46.6)	495 (29.4)		
2 packs	768 (35.1)	657 (39.1)		
3+ packs	399 (18.3)	530 (31.5)	145.3	<0.001
Age 25-34				
1 pack	324 (33.7)	160 (21.2)		
2 packs	354 (36.8)	244 (32.4)		
3+ packs	284 (29.5)	349 (46.3)	57.9	<0.001
Age 35+				
1 pack	550 (32.2)	294 (27.4)		
2 packs	668 (39.1)	328 (30.5)		
3+ packs	492 (28.8)	452 (42.1)	52.9	<0.001

Number of non-pharmacy pack equivalents of pure paracetamol used in overdoses in England (three centres, six general hospitals) (maximum pack size = 16 tablets), and in Ireland (maximum pack size = 12 tablets), 2002 to 2007.

## Discussion

We have used data collected through special registers in six general hospitals in three centres in England and in all general hospitals in Ireland to determine whether the differences in pack sizes of paracetamol sold over the counter in pharmacies and non-pharmacy outlets in the UK and Ireland are reflected in the size of overdoses of paracetamol. There were peaks in the numbers of tablets taken in paracetamol overdoses for both England and Ireland which reflected the maximum pack sizes in the respective countries. These were not only found for the maximum single pack sizes, but also for multiples of these pack sizes. The latter could reflect overdoses where multiple packs were purchased for the acts. The additional peaks in both samples at 10 and multiples of 10 tablets presumably reflect effects of rounding or approximation by patients, and, possibly, clinicians.

There was a marked difference between England and Ireland in terms of the pack equivalents of paracetamol taken in overdoses, based upon the maximum pack size for non-pharmacy and pharmacy preparations in the two countries. More packs were in general consumed in overdoses in Ireland. This difference was found for both genders and across all three age groups that were examined. This raises the following questions: (a) Is advice on sales of packs being followed to the same extent in the two countries? (b) Do purchasing patterns differ, with perhaps paracetamol packs being bought with greater frequency in Ireland, so that more are available in households? (c) Are there differences in patient characteristics between the two countries influencing patterns of self-poisoning? and (d) Are individuals in Ireland who have taken paracetamol overdoses less likely to present to hospital when the amount taken is relatively small? Unfortunately, we do not have access to over-the-counter sales data for the two countries. We have no reason to believe that there are major differences between the characteristics of patients who take paracetamol overdoses in England and Ireland, except that it appears that paracetamol is taken more frequently in overdose in England, the female to male gender ratio is greater in Ireland, and a somewhat greater proportion of patients in Ireland are in the youngest age group. These differences do not, however, appear large enough to explain the extent of the difference in patterns of pack consumption in overdoses. We do not have information in this study on the frequency of hospital presentations in relation to size of overdoses in the two countries. In a recent schools-based study the frequency with which adolescents who reported taking overdoses (often of paracetamol) presented to hospital did not differ markedly between England and Ireland [21-23]. However, one important difference between the two countries is that many individuals who present to emergency departments in Ireland are subject to a fee, whereas this is not the case in England. Also, obtaining general practitioner care in Ireland often involves a fee, which could influence willingness to seek help for emotional problems and therefore risk of self-poisoning. The more rural nature of Ireland, and hence larger distances to hospitals for many residents, could also influence presentation to emergency departments.

An important possible explanation for the difference in number of packs used for overdoses in England and Ireland may be less rigorous enforcement of sales advice contained in Regulatory notices in Ireland compared with England. A study conducted before the Irish legislation was introduced indicated that sales advice for non-pharmacy outlets from the Irish Medicines Board was often not being followed [24]. Following the introduction of the legislation, researchers visiting pharmacy and non-pharmacy outlets in Dublin were able to purchase in excess of statutory limits of paracetamol in a single transaction in half of all pharmacies and the majority of non-pharmacy outlets. This situation largely persisted when the researchers revisited outlets a year later [25]. The present situation in Ireland in this regard requires investigation. There is also evidence of breaching of the legislation in non-pharmacy outlets in England [26] but this appeared to be to a far lesser degree than in the post-legislation study in Ireland [25].

There was only limited evidence that different restrictions in pack sizes in England and Ireland may have had an impact on sizes of overdoses taken in the two countries. While there was no difference when all episodes were considered, smaller paracetamol overdoses were taken in Ireland than England by females aged 35 years and over. Although the difference was small, a relatively minor shift in number of tablets taken in overdoses could have a considerable effect at a population level in terms of numbers of patients developing hepatotoxicity. Whether or not alcohol was involved in the act of self-poisoning made little difference to the result, except for a trend for smaller overdoses to be taken by males in Ireland who did not consume alcohol in association with the act.

### Strengths and limitations

The study involved large numbers of patients in both countries. The Irish sample was based on all overdose presentations to general hospitals in Ireland. However, the English sample was based on presentations to six general hospitals in three centres. It is not known if these are representative of all general hospitals in England, particularly since some of them are in large urban areas and rates of self-harm vary across the centres [18,19]. The overall rates and patterns of self-harm in Ireland and England appear to be similar [18,20,27].

We chose a relatively low amount for defining self-poisoning as some people who intentionally take an overdose only consume a few tablets, even some with suicidal intent [28].

The recording of the number of tablets taken in overdoses relied largely on patient self-report, which is known to be subject to inaccuracy [29]. Also, there was evidence of possible rounding or approximation, with peaks in the numbers of tablets reportedly taken in overdoses in both countries at 10 and multiples of 10 tablets. The peaks seen at numbers of tablets equivalent to the different pack sizes in the UK and Ireland may more accurately reflect sizes of overdose as patients may be more likely to consume whole packets of tablets, especially when these have been purchased for the purpose of self-poisoning.

A further limitation is that we did not have data on suicidal intent [30] for patients from the two countries. Suicidal intent can influence the danger of acts of self-harm [31]. Data on suicidal intent for patients in the two countries could therefore have helped in the interpretation of the findings.

We have restricted our study to pure paracetamol overdoses, with or without co-ingestion of alcohol. We do not know whether the findings would also apply to overdoses of paracetamol compounds which are subject to the same pack size limitations, or those where paracetamol or paracetamol compounds are taken with other substances.

Finally, we do not have data for Ireland on possible changes in size of overdoses following the introduction of the 2001 legislation on pack sizes there [6], although the size of overdose for which calls were made to the National Poisons Centre decreased in the first two years after the legislation was introduced [12]. In England, reductions were found in deaths and in liver transplants due to paracetamol overdose following the 1998 UK legislation [1,7].

## Conclusions

This study has shown that people who take paracetamol overdoses tend to consume numbers of tablets related to available pack sizes. In Ireland, people tended more often than in England to take numbers of tablets equivalent to multiple packs. This raises the question of whether this reflects differences in patients' characteristics, access to care, and greater ease of purchasing multiple packs in Ireland compared to England. The findings suggest that smaller packs of paracetamol sold through Irish pharmacy and non-pharmacy outlets may be associated with smaller overdoses than in England in females aged 35 years and over. However, no differences were found in other age and gender groups. Currently, data on death rates for paracetamol overdoses in Ireland have not been published.

The findings do not provide information on whether a further reduction in the maximum pack sizes for paracetamol sold over the counter in the UK would have further beneficial effects on size of overdoses (and hence on deaths from paracetamol overdose). Further investigation of whether there are differences in enforcement of legislatory advice on sales would be of interest, as would further comparative studies of the apparent effects of the legislation in the two countries on deaths and hepatotoxicity.

## Competing interests

The authors declare that they have no competing interests.

## Authors' contributions

KH conceived of and designed the study, and drafted the manuscript. KH, HB, EA, PC, JC, KW and NK were responsible for data collection. HB conducted the statistical analyses. HB, SS, EA, PC, JC, KW, DG and NK commented on drafts of the manuscript and assisted with interpretation of data. All authors read and approved the final manuscript.

## Pre-publication history

The pre-publication history for this paper can be accessed here:

http://www.biomedcentral.com/1471-2458/11/460/prepub
